# A Hybrid Method for 3D Reconstruction of MR Images

**DOI:** 10.3390/jimaging8040103

**Published:** 2022-04-07

**Authors:** Loubna Lechelek, Sebastien Horna, Rita Zrour, Mathieu Naudin, Carole Guillevin

**Affiliations:** 1 XLIM Laboratory, Joint Research Unit, National Center for Scientific Research (UMR CNRS) 7252, University of Poitiers, CEDEX 9, 86073 Poitiers, France; sebastien.horna@univ-poitiers.fr (S.H.); rita.zrour@univ-poitiers.fr (R.Z.); 2 Common Laboratory Multi-Nuclear Multi-Organ Metabolic Imaging (I3M), CNRS-Siemens, University and Hospital of Poitiers, 86000 Poitiers, France; mathieu.naudin@chu-poitiers.fr (M.N.); carole.guillevin@chu-poitiers.fr (C.G.); 3 LMA Laboratory, Joint Research Unit, National Center for Scientific Research (UMR CNRS) 7348, University of Poitiers, CEDEX 9, 86073 Poitiers, France

**Keywords:** 3D reconstruction, Marching Cubes, implicit models, MR imaging

## Abstract

Three-dimensional surface reconstruction is a well-known task in medical imaging. In procedures for intervention or radiation treatment planning, the generated models should be accurate and reflect the natural appearance. Traditional methods for this task, such as Marching Cubes, use smoothing post processing to reduce staircase artifacts from mesh generation and exhibit the natural look. However, smoothing algorithms often reduce the quality and degrade the accuracy. Other methods, such as MPU implicits, based on adaptive implicit functions, inherently produce smooth 3D models. However, the integration in the implicit functions of both smoothness and accuracy of the shape approximation may impact the precision of the reconstruction. Having these limitations in mind, we propose a hybrid method for 3D reconstruction of MR images. This method is based on a parallel Marching Cubes algorithm called Flying Edges (FE) and Multi-level Partition of Unity (MPU) implicits. We aim to combine the robustness of the Marching Cubes algorithm with the smooth implicit curve tracking enabled by the use of implicit models in order to provide higher geometry precision. Towards this end, the regions that closely fit to the segmentation data, and thus regions that are not impacted by reconstruction issues, are first extracted from both methods. These regions are then merged and used to reconstruct the final model. Experimental studies were performed on a number of MRI datasets, providing images and error statistics generated from our results. The results obtained show that our method reduces the geometric errors of the reconstructed surfaces when compared to the MPU and FE approaches, producing a more accurate 3D reconstruction.

## 1. Introduction

Imaging technologies such as computed tomography (CT) and magnetic resonance imaging (MRI) have been intensively developed and are now commonly used in medicine, science and engineering to study the internal structures of a variety of specimens and organs. In medical assessment, physicians still largely use the individual 2D slices of the 3D volume dataset to evaluate diseases. However, visualizing 3D structures is also very advantageous for understanding the pathophysiological processes. For instance, the study of neurodegenerative diseases such as Alzheimer’s involves the reconstruction of the brain cortex to accurately estimate the cortical thickness [1]. Frequently, the reconstruction process is preceded by the delineation and segmentation of specific regions in the slices. These regions, representing a segmentation map, are then converted into 3D meshes in order to be observed or used for therapeutic planning and other analyses. Many authors have tackled the problem of reconstructing 3D models from medical image data. This subject is motivating for many fields, such as medical diagnosis, prognosis, virtual education and visualization, model-based therapy planning, or surgical planning. One of the core questions for these approaches concerns the smoothness of reconstruction, since anatomical structures usually do not exhibit sharp edges. However, it is also important to generate accurate 3D models that closely approximate the original medical data.

Historically, reconstruction techniques were introduced with the standard Marching Cubes (MC) isosurface extraction algorithm [2]. Conceptually, the MC algorithm divides a 3D volume representing a specific structure into a discrete set of cubes and approximates the mesh going through each cube by a set of zero or more triangles. It is widely used for reconstructing a surface from medical data due to its simplicity, rapidity and its robustness to deal effectively with various types of data, such as isotropic and anisotropic data. The popularity of the MC algorithm and its widespread adoption has led to several improvements in the algorithm to enhance the quality of the generated mesh [3] or to reduce the computational cost [4]. Recently, Schroeder et al. [5] proposed a fast isosurface extraction algorithm called Flying Edges (FE). This algorithm is especially designed to achieve a large scale with low memory requirements. However, since it is based on the standard MC algorithm, it has inherited some particular shortcomings. The generated mesh may contain several artifacts, such as staircases and terraces, since the Marching Cubes mesh closely follows sharp voxel boundaries (instead of smoothly fitting them). Thus, reconstructing smooth curves is still an approximation. Flying Edges (FE) adds an additional mesh smoothing post processing to reduce artifacts from mesh generation, but this may degrade the accuracy since relevant features may be removed and distances between output surfaces and segmentation are changed [6].

Contour-based methods offer an elegant alternative for directly producing smooth 3D models. Most of them are based on the idea of converting the set of contours into 2D distance fields and interpolating these fields using inter-slice interpolation methods [7,8,9]. However, interpolating intermediate slices results in much more data and computational effort. In a different and much faster approach, Braude et al. propose Multi-level Partition of Unity (MPU) implicit models [10]. MPU implicits offer advantages unlike other contour-based reconstruction techniques, since they are based on implicit functions that adaptively conform to local features and details. They are able to produce smooth curves thanks to the use of piecewise local quadratic functions that capture efficiently the local shape of the surface. Nevertheless, this method has some drawbacks. One difficulty is the inclusion in the implicit functions of both the accuracy and smoothness of reconstruction. These two properties are controlled by a single parameter called the tolerance. To achieve a high level of smoothness around some regions, a large value of tolerance has to be used, which will subsequently result in a poor approximation of the original segmentation.

The present work relies on the two following methods: Flying Edges (FE) and Multi-level Partition of Unity (MPU) implicit models. Our main objective is to provide a reconstruction method that fully benefits from the features brought by both of them, and generates a smooth and accurate 3D model that closely approximates the real medical data. In this paper, we first describe each reconstruction method and discuss the key strengths and weaknesses of each of them. We then propose an efficient hybrid approach, in which the regions that closely fit to the segmentation data (pronounced correct regions) are identified and extracted from both methods. These regions are then merged and used to reconstruct the final 3D model. More precisely, the main contributions and advantages of our work can be summarized as:

- A comparative analysis between FE and MPU implicits, which identifies the key strengths and weaknesses of each method;
- A reconstruction process that allows the strengths of one method to offset the weaknesses inherent in another, offering thus a higher geometry precision;
- A reference metric for evaluating the quality of reconstructions, based on a qualitative and quantitative analysis.

We show results on a variety of MRI datasets, demonstrating that our method produces a more accurate reconstruction than the usual methods (FE and MPU). The remainder of this paper is organized as follows: we first discuss the state of the art (Section 2), before describing the two reconstruction methods, FE and MPU (Section 3). We then introduce the principle of our 3D hybrid reconstruction method (Section 4) and discuss the results of the three methods, tested with a number of MRI datasets (Section 5). Finally, we conclude with an outlook on future works (Section 6).

## 2. Related Work

The 3D reconstruction of objects has received much interest in a wide variety of fields, such as computer graphics and computer vision, computer animation, medical imaging, virtual reality, or computational science. This paper focuses on the 3D reconstruction methods used in the medical field and more particularly on the reconstruction methods applicable to MRI images. Historically, they have been introduced with the standard Marching Cubes (MC) isosurface extraction algorithm [2]. The simplicity, robustness and rapidity of the Marching Cubes have made it the most widely used method for the interactive visualization of medical data. However, the generated mesh may present discontinuities and topological incoherences due to the ambiguities in the interpolant behavior [11,12]. This is the reason why several authors have increased the number of entries in the Marching Cubes triangulation lookup table [13,14,15,16] to resolve these ambiguities and thus enhance the topological correctness [17,18]. Unfortunately, the reconstruction of a precise geometric mesh that reflects properly the anatomical structure still is an active research field, since the extracted mesh often contains degenerate and skinny triangles [3,19]. Furthermore, meshing is often constructed from a 3D volume defined in the discrete space and the process hardly reconstructs smooth curves. This lack of precision leads to artifacts such as staircases and terraces, leading to imprecise meshes that do not reflect the natural appearance of anatomical structures. Recently, Schroeder et al. [5] introduced the Flying Edges, which is the parallel version of the Marching Cubes. This algorithm is now implemented in the medical visualization and analysis platform 3D slicer, with an additional mesh smoothing post processing. Smoothing algorithms allow us to reduce artifacts from mesh generation and achieve a natural appearance since the anatomical structures usually do not exhibit sharp edges, but in turn it may reduce the quality and degrade the precision [6]. In particular, this may alter the structure in some regions since the distance between the mesh and the original segmentation is changed.

Moreover, numerous contour-based surface reconstruction techniques have been developed, either with explicit or implicit surface representations. Contour stitching methods interpolate between the adjacent cross-sectional contours using a mesh, composed of straight lines and flat faces [20,21,22,23,24,25]. However, representing a mesh by only a set of straight lines does not allow one to depict smooth biological data. In addition, these early approaches fail to deal effectively with handling special cases such as keyholes contours, rapid changes, and branching. These issues have been addressed recently by Sunderland et al. [26] in a more robust algorithm for interpolating planar contours. However, despite all the improvements, this method still has two major drawbacks: the resulting mesh is not topologically correct (i.e., it might contain triangles that converge to a single point at the edge of the contour) and it tends to produce a high number of triangles.

In contrast, methods based on implicit surface representation provide a more stable surface reconstruction, since they maintain the smoothness of the biological structures, regardless of the complexity of the regions. A first approach in this category interprets the cross-sectional contours as a stack of smooth and continuous 2D distance fields. These latter are then interpolated in the z-direction using inter-slice interpolation methods [7,8,9,27,28,29]. Although these methods produce smooth 3D models, the main drawback is that the interpolated results may be affected by the disturbance of scanning noise and segmentation errors on medical images, thus reducing the precision of the generated mesh. Another approach tends to fit an implicit function to the input data using provided normals, after which the final surface can be obtained by extracting an isosurface. A prominent example includes the Multi-level Partition of Unity (MPU) implicit models [10], inspired by the work of Ohtake et al. [30]. We have chosen, in the current work, to build upon the Flying Edges [5] and MPU implicit approach [10] and describe them in detail in Section 3.

## 3. Flying Edges and MPU Implicit Approach

**Figure 1:**
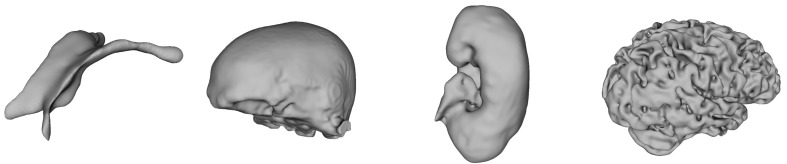
Three-dimensional reconstructed models from MR images used for our study.

This section details the two reconstruction methods, Multi-level Partition of Unity (MPU) implicit models and Flying Edges (FE), used in our hybrid reconstruction system. The 3D models illustrated in Figure 1 have been used to study the behavior of the two reconstruction algorithms (MPU and FE). Additional case studies from complex real-world data are presented in Section 5.

### 3.1. Flying Edges (FE)

**Figure 2:**
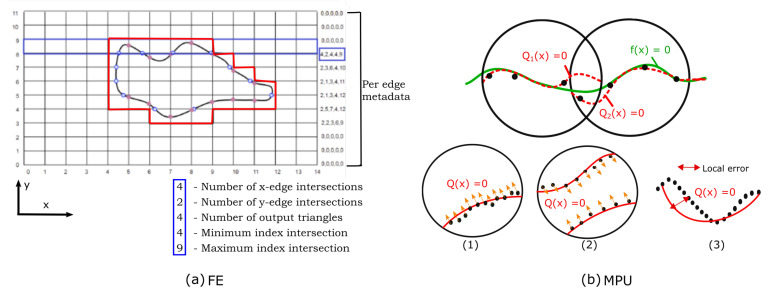
FE and MPU: (**a**) The region of the FE grid processed at the end of the second stage; on the right side is a metadata array describing the interaction of the isosurface with the grid edges. (**b**) Principle of MPU. Top: Two local approximations (dashed red curves) blended to form the global MPU function (solid green curve). Bottom: (1–2) quadric functions are used to approximate the local shape; (1) bivariate quadric is used; (2) general 3D quadric is used; (3) the local function’s error is evaluated in each step of the algorithm in order to determine if the local function has to be refined.

**Figure 3:**
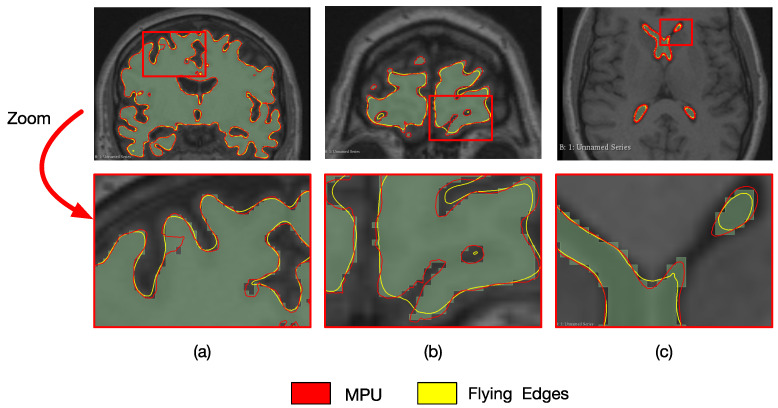
Reconstruction of the brain and ventricle with FE (in yellow) and MPU (in red); (**a**,**b**) coronal brain cross-sections; (**c**) axial ventricle cross-section; (top) overlaid models; (bottom) a zoom on the red rectangle showing the reconstruction results of each method.

The Flying Edges (FE) was designed from the standard MC algorithm with parallelism in mind [5]. It operates on a 3D grid of voxels and uses a lookup table from which the triangle configuration of each voxel is determined. Instead of marching through all the voxels in a single pass, FE uses multiple preprocessing passes to guide and reduce dramatically subsequent computations. The first preprocessing stage marches through all the voxels along one dimension of the volume and determines which edges are intersected. The second stage determines which edges along the other two dimensions are intersected using information gained in the first preprocessing stage. The sections are crossed in parallel and each indicates its first and last intersections, so that only a smaller part of the grid will be processed, thus reducing the time to find connections between triangles generated. For example, as shown in Figure 2a, after the second pass, the grid (in 2D) is restricted to a small region circumnavigating the isosurface. The third of the preprocessing stages allocates memory to contain the triangles and points, while the last stage calculates the intersection points and generates triangles. Moreover, 3D slicer [31] uses VTK’s implementation [32] for FE and adds an additional mesh smoothing post-processing step [33] to remove staircase artifacts and to exhibit the natural look of anatomical structures. We have chosen this algorithm for its robustness to deal effectively with various types of data. In addition, it is computationally efficient and scales favorably for large datasets. However, the generated mesh may be impacted by the inherent issues of the Marching Cubes algorithm, coming essentially from the triangulation lookup tables [3] and the smoothing post processing [6]. FE outputs 3D models that are geometrically smoothed using a smoothing filter. As a result of surface smoothing, artifacts are reduced but, as a side effect, the distance between the output surface and the original segmentation might be altered too much in some regions, reducing the accuracy of reconstruction (Figure 3b,c).

### 3.2. Multi-Level Partition of Unity (MPU) Implicit Models

The Multi-level Partition of Unity (MPU) implicit models presented in [10] operate on a set of contours, representing the outlines of the structure, and produce a smooth 3D mesh as output. The coordinates of the contour pixels (x,y), along with the slice number, allow us to convert the set of contours into a set of 3D points in $R^{3}$. The points are subdivided using a recursive octree-based subdivision scheme, where the surface estimation of each partition is performed locally and the overlapping local implicit functions are blended together to produce the overall surface (i.e., zero iso-surface, from which the mesh is then extracted). Figure 2 briefly recalls the principle. Since the MPU requires surface normal information, the surface normal at each contour point is estimated from the binary volume, constructed from the set of contours as described in [10]. The implicit functions are approximated using local quadric functions, where the choice of the appropriate function depends on local surface features implied by the point normals (Figure 2b(1) ) and (Figure 2b(2)). At each stage of the subdivision process, the local function’s accuracy has to be evaluated and compared to a user-specified tolerance value (tol) in order to determine if the local implicit surface needs to be refined (Figure 2b(3)) and thus allows us to refine areas of higher detail.

We have chosen to build upon this method, because the resulting mesh is not geometrically smoothed through a post processing as in Flying Edges. Smoothing is basically included in the reconstruction process, since the MPU is based on mathematical implicit functions that smoothly conform to the local features and details of structures. However, the flexibility of specifying the smoothness and the accuracy of the shape approximation are both controlled by the tolerance parameter tol. To achieve a high level of smoothness around the points, when fitting the implicit function to the target structure, a relatively large value of the tolerance has to be used, which will subsequently result in a poor approximation of the original geometric shape of the structure, especially in the case of non-dense datasets or objects with intricate geometry (Figure 3a).

The reconstruction results and visualization of regions with errors are presented in Figure 3. The models are overlaid with two different colors to see them together, and a zoom on a specific zone (red square) is included to show the reconstruction differences. One can observe that reconstruction results may differ from one cross-section to another and/or from one structure to another. For example, the cross-section of the brain in Figure 3a shows that FE better follows the segmentation when compared to MPU, which is not the case for the cross-section in Figure 3b. Looking at the ventricle in Figure 3c, one can remark that MPU is more precise than FE. Additional results and analysis based on numerous examples are provided in Section 5 and Appendix B. The method that we propose in this paper aims at reducing the drawbacks of both approaches. Our idea is to combine the advantages of each reconstruction method in order to produce a higher geometry precision model, which better preserves the details and the local characteristics of the surfaces. The next section exposes the proposed method, shows how the imprecise 3D regions are localized, and describes our choices for selecting the correct ones.

## 4. 3D Hybrid Reconstruction Method

**Figure 4:**
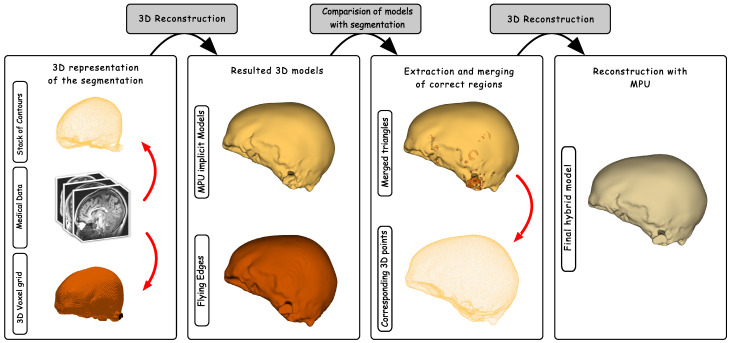
The pipeline of our hybrid reconstruction system: two reconstructed 3D models based on FE and MPU are generated. The resulted models are compared with the segmentation and the correct regions are extracted and merged. The MPU is fit to the points extracted from the merged triangles to produce the 3D geometric mesh.

The goal of the hybrid reconstruction method is to produce a 3D model as close as possible to the data provided by the segmentation process. The main idea is to compare the models produced by the two reconstruction methods, FE and MPU, in order to extract the regions that are more faithful to the segmentation. This is accomplished by computing a distance metric that retrieves from the two models the regions that closely fit to the input structure, and thus the regions that are not impacted by the reconstruction issues. Since the two models will be compared to the segmentation, they should be precisely aligned with the segmented structure in the 3D space. Each 3D model is represented by a triangular mesh (i.e., set of triangles), while the segmentation is represented by a set of 3D points (i.e., the voxel centers of the segmentation boundary within the volume). The principle of our hybrid reconstruction system is illustrated in Figure 4 and it relies on the following stages.

The first stage takes as input the segmentation data and generates the corresponding 3D models, FE and MPU. The second stage compares the 3D models with the segmentation by searching for each triangle in the MPU model $T_{MPU}$, the corresponding closest triangle lying on the FE surface $T_{FE}$. It also searches for the closest 3D point *P* lying on the segmentation.

The distance between the center of each triangle and the point *P* is then computed and the triangle with the smallest distance is retained, as shown in the equation below:

(1) $$\delta \left(T_{MPU},P,T_{FE}\right)=\min(||C_{MPU}-P||,||C_{FE}-P||)$$

where $\delta (T_{MPU},P,T_{FE})$ is the minimum distance between the triangles and the point *P*. $C_{MPU}$ and $C_{FE}$ are the respective centers of triangles of $T_{MPU}$ and $T_{FE}$.

The collection of triangles retrieved from the MPU and FE models is then merged and used to reconstruct the final 3D model. The last stage of the algorithm recovers the 3D points from the set of triangles and approximates the surface defined by the point set with the MPU implicit models. A merging step has to be applied before reconstructing the final surface because an entire region composed of a set of triangles might be recovered from FE or MPU, and thus the points shared with the neighboring triangles should be merged, to avoid the appearance of redundant points in the resulting point cloud. It should also be noted that it is not necessary to compute point normals since they can be directly recovered from the 3D models.

We have chosen to reapply the MPU implicits to reconstruct the final surface for many reasons: they are both space- and time-efficient, are able to deal with unstructured points that vary in sampling density, do not require input points to lie on a plane, and do not need any mesh post-processing (smoothing), which may degrade the accuracy of the resulting 3D model. In addition, since the point cloud extracted from the merged triangles is denser, compared to the points extracted from the set of contours, a very small tolerance value is sufficient to provide a good approximation with a high degree of smoothness.

## 5. Hybrid Reconstruction Results and Analysis

The overall system exposed in the previous sections has been implemented as a plugin module for the 3D slicer platform. All software and code used are written in C++ using the VTK Library [32] (i.e., an open-source software for manipulating and displaying geometric data). We have tested and evaluated our reconstruction technique with a variety of MRI datasets. Table 1 presents detailed information about four of the datasets (Brain, Skull-Bone, Ventricle, and Kidney), including the number of slices, in-plane image resolution, and the ratio of in-plane to slice sampling rates. The first three datasets are included in 3D slicer, while the Kidney dataset is provided by the CHU of Poitiers.

We also evaluated our technique on 39 human healthy brain MRI samples, also provided by our clinical partner. All images are acquired on a Magnetom Skyra 3 Tesla (Siemens Healthineers, Erlangen, Germany). The sequence used is a 3D T1 MPRAGE 0.9 mm isotropic (Te = 2.41 ms, Tr = 1950 ms, Ti = 816 ms, FOV = 256 × 213 mm${}^{2}$, matrix: 240 × 288, slices: 192, turbo factor: 224). Original DICOM data are converted to NIFTI to be used in our homemade automated pipeline. As post-processing, FSL-BET [34], provided as a part of the FSL software (http://www.fmrib.ox.ac.uk/fsl/ (accessed on 20 November 2020)) package, is applied to remove subcutaneous fat in order to create a mask of brain only. Then, FSL-FAST [35] is launched to obtain the three segmentation classes: (i) cortex, also known as grey matter; (ii) white matter; (iii) cerebral spinal fluid.

The reconstruction quality was examined in detail through analysis and error measurements of each reconstructed model. Additionally, we investigated the effectiveness of our method through analyzing the curvature of the reconstructed surface and compared the results from our approach to those produced by FreeSurfer (https://surfer.nmr.mgh.harvard.edu/ (accessed on 15 March 2021)) [36].

### 5.1. Evaluating Reconstruction Error

**Figure 5:**
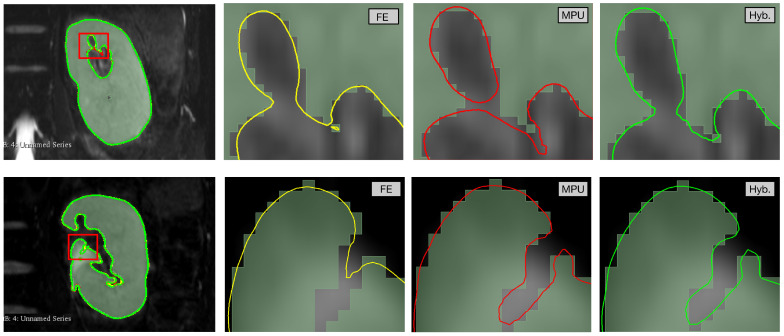
A visual evaluation of the Kidney model, reconstructed with FE (yellow), MPU (red), and Hybrid (green). From left to right: overlaid models followed by a zoom on the highlighted areas showing the reconstruction result of each method.

Figure 5 provides a detailed visual comparison between the three approaches. We focus on the areas highlighted by the red block in different colors. On the top row, the MPU (in red) constructs two separate components, while FE (in yellow) finds one main component with a small noisy region. The hybrid (in green) performs better, since it reconstructs one component, following closely the segmented region. On the bottom row, the MPU is more precise than FE and the hybrid recovers the region constructed by the MPU since it is still the closest one to the segmentation. It is noted that our hybrid method achieves a correct reconstruction in all highlighted areas.

Although the visual evaluation of the reconstructions is reliable, it is necessary to quantify the quality of the reconstructions in order to determine how faithfully the 3D models fit to the segmented structure. Two distance-based metrics are used to estimate the geometric errors: absolute distance and Hausdorff distance [37]. The absolute distance measures the distance between each point of the segmentation and the nearest point of the 3D model. The distance values are then gathered and statistics related to the complete reconstruction are calculated. The Hausdorff distance [37] computes the maximum distance between two sets of sampled points from the segmentation and the reconstructed surface. All distances are computed bidirectionally in millimeters (mm) and represented as 3D color-coded maps, where reconstruction errors are displayed from blue (low) to red (high).

**Figure 6:**
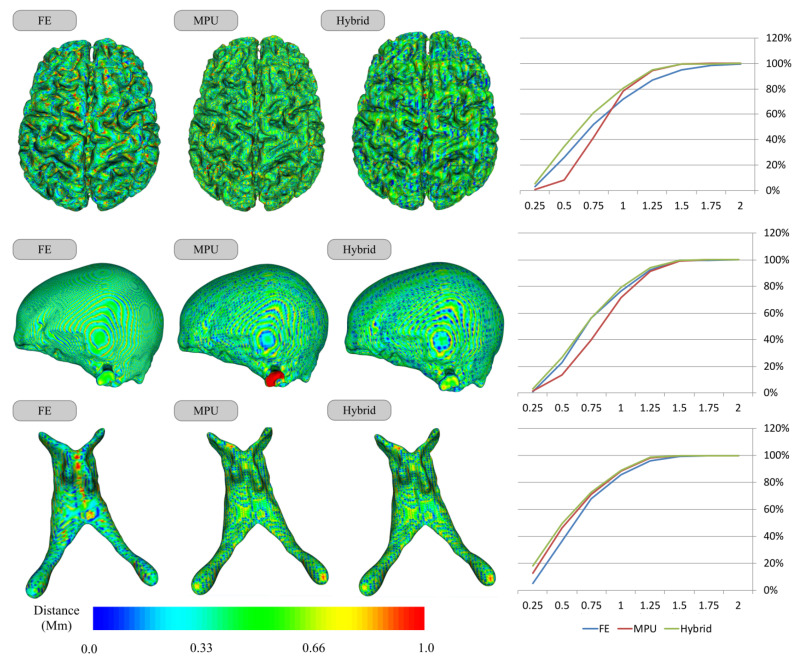
Distance from the segmentation for the reconstruction methods: FE, MPU, and Hybrid. The voxel size of the segmentation grid in XY dimension is of 1 mm; (**left**) reconstruction error using Hausdorff metric; (**right**) cumulative percentage of surface points as a function of their distance to the segmentation.

Figure 6 presents the results of the distance measure on the Brain, Skull-Bone, and Ventricle models for each of the three reconstruction methods. For each model, by examining the Hausdorff color-coded reconstruction maps, one can remark that, on average, the reconstructions are around 1 mm away from the segmentation, which is manifested by the predominance of the green color, as shown on the left side of Figure 6. The Hausdorff color maps show also the efficiency of our hybrid method, which retrieves the correct regions (i.e., the closest ones to segmentation) from FE and MPU. For instance, the Skull-Bone color maps show the presence of a red area (i.e., a region that has an error greater than 1 mm) on MPU, which does not appear when moving to FE, where this flat area is perfectly reconstructed. Our method recovers thus the FE area since it is closer to segmentation. Looking at the curves on the right side, the statistics show that for all models, our hybrid performs better than FE and MPU since it is still the closest one to the segmentation. If we consider small distances, it has the greater percentage of points, since it retrieves the closest regions either from FE or from MPU, offering 3D models benefiting from the accurate reconstruction of the flat regions allowed by the FE and the smoothed contour tracking of high curvature regions allowed by the use of implicit models.

The minimum, maximum, arithmetic mean, and the standard deviation of the distance values for each reconstruction method are given in Table 2, as well as the percentage of points that are one and half voxel sizes away from the segmentation. All of the metrics that are presented here are in voxel units (1 mm). For example, a maximum distance of 2 signifies that the point surface lies, at most, 2 voxels away from the segmentation. Once again, we can remark that, for all models, while the maximum distance values for the hybrid lie between 1 and 4 voxels, the means and standard deviations still stay relatively low. In addition, the percentage of points indicates that our hybrid still has the greater percentage of points (around the voxel size of 1 mm) compared to FE and MPU.

**Figure A1:**
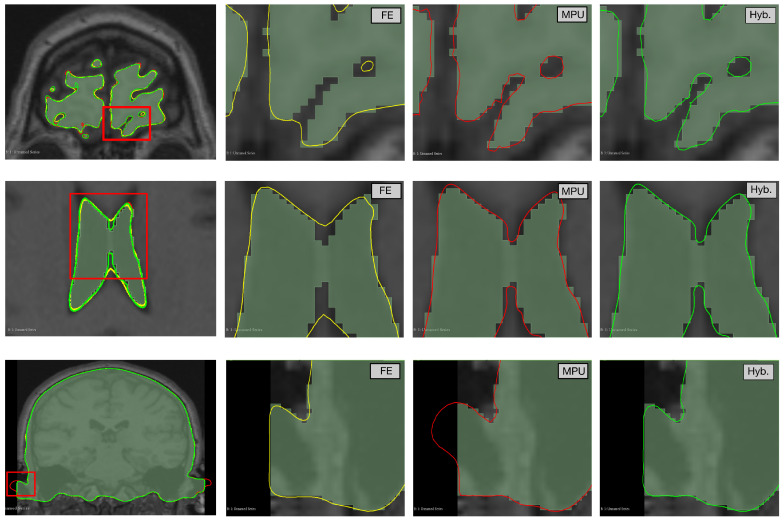
Reconstruction of the Brain, Ventricle, and Skull-Bone with FE (yellow), MPU (red), and Hybrid (green). From left to right: overlaid models followed by a zoom on the highlighted areas showing the reconstruction result of each method.

We also evaluated the effectiveness of our hybrid method on the 39 human healthy brain MRI samples. The statistics related to each reconstruction method are given in Table 3 and Figure A1.

**Figure 7:**
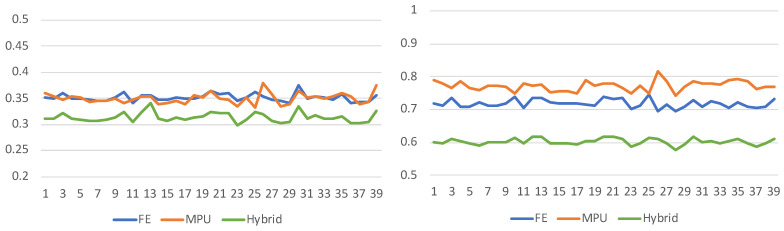
The standard deviation (**left**) (on average: Hybrid—0.31, FE—0.35, MPU—0.35) and arithmetic mean (**right**) (on average: Hybrid—0.60, FE—0.71, MPU—0.77) of the error values for each of the 39 cerebral cortices presented in the Table 3 and Appendix A, and reconstructed with FE, MPU, and Hybrid. The hybrid method has a lower standard deviation and arithmetic mean.

One can remark that the maximum distance values for the FE lie between 2 and 3 voxels, compared to the MPU and the hybrid, where the maximum distance value can reach 8 voxels for some cortices. However, the percentage of points indicates that almost all regions of the hybrid model lie within the voxels defined by the segmentation. On average, 88.56% of the surface points for the hybrid are around the voxel size of 1 mm, followed by MPU with 80.51% and FE with 78.39%. This demonstrates that the reconstruction error of the hybrid is sub-voxel for the vast majority of surface points. If we consider small distances (less than 0.5 mm), the hybrid performs even better, since 45.23% of the points are less than half a voxel size, versus 31.18% for the FE and only 20.31% for the MPU. Results can be further deepened looking at the standard deviations (Figure 7), where the statistics show that the hybrid still stays relatively low compared to FE and MPU. Moreover, looking into the arithmetic mean, the hybrid has the lower one, followed by the FE and then MPU.

### 5.2. Curvature Analysis

**Figure 8:**
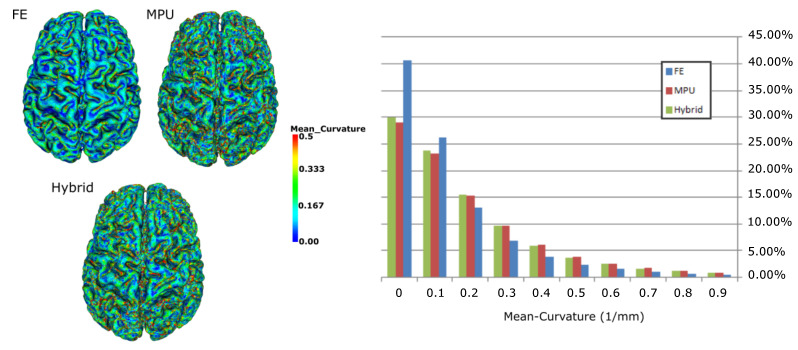
Visualization of the mean curvature on a 3D brain model: (**left**) triangles are colored depending on their curvature value (blue reflects low curvatures and red high curvatures); (**right**) percentage of points as a function of their mean curvature value.

Besides the qualitative study exposed above, we also evaluated quantitatively the reconstruction methods through examining the curvature variation of the reconstructed surfaces. The curvature behavior conveys rich information about the intrinsic shape and provides some insight about the amount of smoothness of the underlying surface. Figure 8 presents the results of the measure of the magnitude of the mean curvature on each point of the brain model for each of the three reconstruction methods. On the left side of the Figure, one can observe that the blue-green faded color is predominant for the three models, while the red color (very high curvature) is much less dominant for the FE compared to MPU and hybrid. This means that, overall, the curvature surface of the FE is relatively low compared to MPU and hybrid. Results can be validated looking at the statistics on the right side of Figure 8, which shows the mean curvature versus the percentage of points. If we consider low curvatures (between 0.0 and 0.2), the FE has the greater percentage of points; however, this discrepancy vanishes with higher curvatures, where the FE has the smaller percentage of points while MPU and hybrid are very close. This is mainly due to the smoothing post processing applied on the output FE models, which tends to change the local surface shape and oversmooth areas with high curvatures. It should be noted that since the brain surface is highly folded, flat curvatures must not be predominant. As for MPU, it appears to better maintain the local surface shape of the model, since it is based on adaptive implicit functions that confine the reconstruction to a specified tolerance parameter, and the amount of smoothing applied to the data is still controllable. The hybrid retains this advantage and ensures the accuracy and smoothness of reconstruction while respecting the local surface shape.

### 5.3. Comparison to FreeSurfer

**Figure 9:**
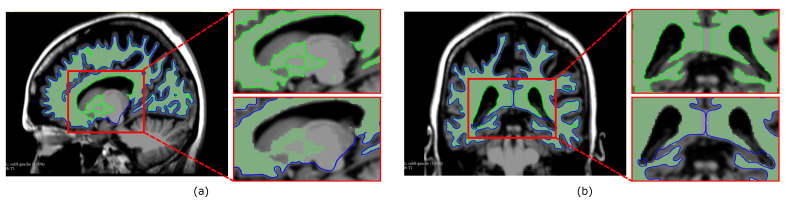
Two examples of the reconstruction of the white matter of the brain with our method in green and FreeSurfer in blue. (**a**) FreeSurfer surface is not properly reconstructed, some details are missing. (**b**) FreeSurfer is unable to push enough the surface area through the narrow opening.

**Figure 10:**
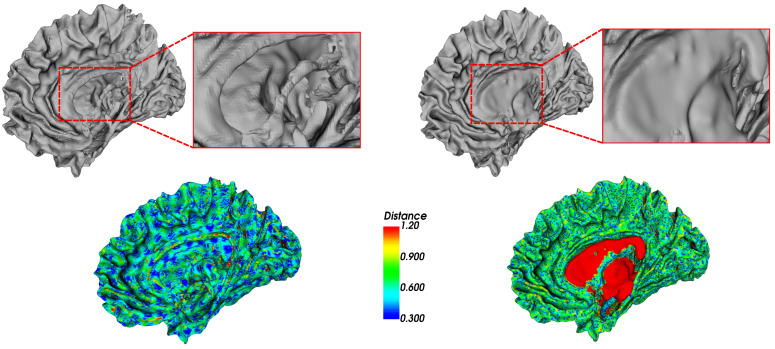
Visualization of the white matter surfaces reconstructed with our hybrid on the left side and FreeSurfer on the right side. For each reconstruction method, on the top, a 3D view is shown, with a zoom on the red rectangle of the reconstruction result. On the bottom, the surfaces are color-coded with the absolute distance to the segmentation from blue (small distance) to red (high distance) using the Hausdorff metric.

Figure 9 and Figure 10 illustrate a comparison between our hybrid method and the reconstruction method used by FreeSurfer (https://surfer.nmr.mgh.harvard.edu/ (accessed on 15 March 2021)) [36] (i.e., an open-source software suite for processing and analyzing (human) brain MRI images). The reconstruction is launched using the FreeSurfer command “recon-all” that performs all the FreeSurfer cortical reconstruction process, including the preprocessing steps. The results show that our method achieves better geometric accuracy when compared to FreeSurfer. It generates surfaces that accurately track the borders of the white matter, compared to FreeSurfer, which ignores some regions. The FreeSurfer reconstruction method is based on a deformable surface approach that is typically driven by an energy functional designed to move it towards the segmented structure [38]. The main issue of this approach lies in the difficulty of pushing the surface through the many narrow openings into deep sulci, as illustrated in Figure 9, missing some relevant details. For example, Figure 10 shows that FreeSurfer did not reconstruct correctly the inner regions and attempted to close the exiting hole in the white matter. This is manifested by the red areas (i.e., areas with high distance to the segmentation boundary) presented on the FreeSurfer Hausdorff color map, unlike our hybrid model, which remains closer to the segmentation.

### 5.4. Runtime

We computed the runtime for each of the 39 brain cortices, for each reconstruction method. Flying Edges (FE) and MPU achieve the fastest runtime (<10 s). Our hybrid model takes, on average, <40 s as it applies the two methods and requires additional processing to select the correct regions from the FE and MPU models.

## 6. Conclusions and Perspectives

In this work, we have proposed a hybrid reconstruction method that combines the Multi-level Partition of Unity (MPU) with Flying edges (FE). It performs an extraction of the correct regions (i.e., the most faithful to the segmentation) from both methods, before merging them to reconstruct a final accurate 3D model. This hybrid approach overcomes thus the disadvantages of each of the two reconstruction methods when the reconstructed region is further from the segmentation. We evaluated the effectiveness of our hybrid method on 39 human MRI healthy brain images. The hybrid approach generates a smooth surface model and seems more precise, since it offers sub-voxel accuracy to the segmentation, producing thus that a lower standard deviation and arithmetic mean when compared to FE and MPU. We also evaluated quantitatively the curvature variation of the reconstructed surfaces. The hybrid preserves better the details and the local characteristics of the surfaces compared to FE and MPU. We also compared our method to FreeSurfer [10] and showed that our method generates surfaces that accurately track the borders, compared to FreeSurfer, which ignores some regions.

Finally, we have tested our approach on a large number of datasets provided by our clinical partner, and it has been shown that our method seems to be more robust since it generates accurate reconstructions in every case and produces superior results in comparison with other techniques.

One of the possible extensions of this work would be to include metabolite quantification thanks to Magnetic Resonance Spectroscopy to improve the 3D reconstructed model. The final purpose is to have an accurate and precise 3D model that meets medical expectations and that can be used for diagnosis and virtual biopsy. In the long term, a precise reconstruction will offer a non-invasive technique to detect and localize the presence of a tumor, to classify its nature, and to study the tumor metabolism in order to evaluate the response to possible therapies, thereby helping clinicians to optimize the treatment.

## Figures and Tables

**Table 1:** Characteristics of input datasets.

Name	# of Slices	Resolution	xy:z
Brain	65	256 × 256	1:1.3
Skull-Bone	65	256 × 256	1:1.3
Ventricle	65	256 × 256	1:1.3
Kidney	80	384 × 282	1:1

**Table 2:** Approximation quality corresponding to each of the four reconstructed models (Brain, Ventricle, Skull-Bone, and Kidney). Metrics are calculated in units of voxels (mm).

Name	FE						MPU						Hybrid					
	**Min**	**Max**	**St** **dev**	**Mean**	**% <** **0.5**	**% <** **1.0**	**Min**	**Max**	**St** **dev**	**Mean**	**% <** **0.5**	**% <** **1.0**	**Min**	**Max**	**St** **dev**	**Mean**	**% <** **0.5**	**% <** **1.0**
**Brain**	0.008	2.68	0.31	0.79	39.86	72.08	0.006	3.72	0.32	0.81	22.34	81.48	0.01	3.45	0.27	0.69	43.36	84.04
**Ventricle**	0.05	1.78	0.29	0.64	42.59	84.21	0.06	3.34	0.34	0.79	55.13	86.55	0.03	1.53	0.28	0.61	58.52	88.83
**Bone**	0.02	1.04	0.30	0.75	36.67	80.59	0.05	4.73	0.33	0.83	22.64	78.57	0.02	1.53	0.28	0.72	39.51	83.32
**Kidney**	0.01	2.18	0.32	0.76	24.42	74.09	0.02	4.67	0.33	0.80	26.52	76.24	0.008	2.73	0.31	0.66	37.47	85.54

**Table 3:** Approximation quality of reconstructed surfaces for 10 brain cortices. Metrics are calculated in units of voxels (mm).

Sub	FE						MPU						Hybrid					
	**Min**	**Max**	**St** ** dev**	**Mean**	**% <** ** 0.5**	**% <** ** 1.0**	**Min**	**Max**	**St** ** dev**	**Mean**	**% <** ** 0.5**	**% <** ** 1.0**	**Min**	**Max**	**St** ** dev**	**Mean**	**% <** ** 0.5**	**% <** ** 1.0**
1	0.005	2.51	0.35	0.71	32.78	78.41	0.003	7.36	0.36	0.78	19.33	78.73	0.007	3.89	0.31	0.60	45.16	88.64
2	0.010	2.47	0.34	0.71	33.17	78.96	0.011	4.66	0.35	0.77	20.09	79.89	0.010	4.45	0.31	0.59	45.92	88.95
3	0.006	2.41	0.35	0.73	31.28	76.48	0.005	4.81	0.34	0.76	20.63	81.12	0.006	5.06	0.32	0.61	44.76	87.79
4	0.002	2.42	0.34	0.70	33.97	79.32	0.009	5.01	0.35	0.78	19.22	79.10	0.006	5.47	0.31	0.60	44.15	88.64
5	0.007	2.38	0.34	0.70	33.70	79.31	0.092	5.82	0.35	0.76	20.13	81.35	0.003	4.20	0.30	0.59	45.52	89.08
6	0.009	2.42	0.34	0.72	32.11	78.52	0.002	4.96	0.34	0.76	21.04	81.33	0.008	4.10	0.30	0.59	46.69	89.25
7	0.006	2.35	0.34	0.71	33.26	79.13	0.002	5.49	0.34	0.77	20.10	80.68	0.001	3.21	0.30	0.60	44.15	88.64
8	0.006	2.35	0.34	0.71	33.26	79.13	0.002	4.62	0.34	0.77	20.10	80.69	0.001	6.63	0.30	0.60	44.85	88.75
9	0.007	2.49	0.34	0.69	34.89	80.57	0.005	4.99	0.33	0.74	22.11	83.12	0.004	3.85	0.30	0.57	48.60	90.19
10	0.003	2.56	0.34	0.70	34.16	80.12	0.011	4.62	0.33	0.74	21.63	82.75	0.003	3.92	0.29	0.58	46.89	90.01

## Data Availability

Data are provided by the CHU of Poitiers. The data are not publicly available since the owner has not shared datasets in public.

## Appendix A

**Table A1:** Approximation quality of reconstructed surfaces for 29 brain cortices. Metrics are calculated in units of voxels.

Sub	FE						MPU						Hybrid					
	**Min**	**Max**	**St** **dev**	**Mean**	**% <** **0.5**	**% <** **1.0**	**Min**	**Max**	**St** **dev**	**Mean**	**% <** **0.5**	**% <** **1.0**	**Min**	**Max**	**St** **dev**	**Mean**	**% <** **0.5**	**% <** **1.0**
11	0.011	2.55	0.35	0.71	32.71	78.35	0.010	4.25	0.34	0.77	20.45	80.59	0.006	3.80	0.31	0.60	45.74	88.62
12	0.003	2.38	0.36	0.73	31.33	76.29	0.012	4.34	0.34	0.74	21.75	82.89	0.005	4.53	0.32	0.61	44.36	87.50
13	0.002	2.37	0.34	0.70	33.30	79.88	0.009	5.39	0.34	0.78	19.51	79.64	0.006	3.49	0.30	0.59	45.14	89.19
14	0.004	2.56	0.35	0.73	31.18	76.51	0.011	4.18	0.35	0.77	20.47	80.73	0.007	4.51	0.32	0.61	43.69	87.43
15	0.009	2.53	0.35	0.73	31.36	76.56	0.004	5.09	0.35	0.77	20.22	80.33	0.006	8.25	0.34	0.61	43.61	87.65
16	0.011	2.44	0.34	0.72	32.24	78.32	0.010	4.97	0.33	0.75	21.51	82.49	0.009	3.87	0.31	0.59	45.78	88.78
17	0.015	2.37	0.34	0.71	32.31	78.57	0.008	7.20	0.34	0.75	21.22	82.10	0.004	3.79	0.30	0.59	45.72	89.05
18	0.015	2.45	0.35	0.71	32.83	78.32	0.009	5.24	0.34	0.75	21.57	82.14	0.006	4.20	0.31	0.59	46.61	88.74
19	0.016	2.46	0.35	0.71	32.63	78.39	0.010	5.16	0.33	0.74	21.88	82.76	0.007	3.59	0.31	0.59	47.02	89.16
20	0.003	2.33	0.34	0.71	33.07	78.67	0.017	5.02	0.35	0.78	19.35	78.81	0.004	3.88	0.31	0.60	44.99	88.52
21	0.002	2.56	0.35	0.71	34.06	78.94	0.003	4.20	0.35	0.77	20.41	80.39	0.004	5.17	0.31	0.60	45.16	88.50
22	0.007	2.46	0.36	0.73	31.42	75.97	0.010	7.27	0.36	0.78	20.02	79.68	0.009	4.79	0.32	0.61	43.70	86.97
23	0.009	2.48	0.35	0.73	31.70	76.86	0.010	4.38	0.35	0.78	19.81	79.87	0.031	5.50	0.32	0.61	43.72	87.41
24	0.005	2.46	0.36	0.73	31.60	76.51	0.014	4.40	0.34	0.76	20.87	81.13	0.006	5.87	0.32	0.60	45.29	87.70
25	0.005	2.39	0.35	0.71	33.35	78.87	0.011	4.27	0.35	0.77	20.37	80.39	0.009	4.17	0.30	0.59	46.17	88.96
26	0.015	2.42	0.36	0.74	30.70	75.65	0.004	4.30	0.33	0.74	21.58	83.03	0.008	3.97	0.32	0.61	44.09	87.39
27	0.006	2.44	0.35	0.69	35.98	80.32	0.014	6.91	0.37	0.81	18.07	76.31	0.009	4.48	0.31	0.61	43.55	87.99
28	0.015	2.53	0.34	0.71	32.72	78.73	0.014	4.88	0.35	0.78	19.54	78.00	0.006	3.78	0.30	0.59	45.62	89.01
29	0.015	2.54	0.34	0.70	33.24	79.92	0.012	3.87	0.33	0.76	20.22	80.80	0.006	4.16	0.30	0.59	45.69	89.47
30	0.008	2.47	0.37	0.72	33.57	76.42	0.009	5.28	0.36	0.78	19.77	78.76	0.004	7.43	0.33	0.61	44.35	86.92
31	0.007	2.48	0.34	0.70	33.60	79.36	0.003	4.10	0.35	0.78	19.72	79.62	0.006	4.73	0.31	0.60	45.28	88.91
32	0.009	2.41	0.35	0.72	32.50	77.78	0.005	5.42	0.35	0.77	20.03	79.65	0.008	5.72	0.31	0.60	45.24	88.24
33	0.012	2.32	0.35	0.71	32.60	78.42	0.011	4.34	0.35	0.77	20.01	80.04	0.009	3.59	0.31	0.59	45.71	88.84
34	0.012	2.58	0.34	0.70	34.18	79.50	0.003	4.84	0.35	0.78	19.08	79.75	0.005	5.01	0.31	0.60	44.66	88.64
35	0.002	2.43	0.35	0.72	32.87	78.03	0.007	4.19	0.36	0.79	19.14	78.38	0.009	3.46	0.31	0.61	43.80	87.95
36	0.010	2.46	0.34	0.70	33.18	79.65	0.002	4.24	0.35	0.78	19.39	79.25	0.011	4.50	0.30	0.59	45.38	89.44
37	0.010	2.48	0.34	0.70	33.66	79.68	0.009	4.23	0.33	0.76	20.91	81.35	0.017	6.50	0.30	0.58	47.30	89.85
38	0.012	2.49	0.34	0.70	33.25	79.65	0.006	4.02	0.34	0.76	20.11	80.84	0.007	3.80	0.30	0.59	45.46	89.25
39	0.009	2.54	0.35	0.73	31.53	77.09	0.004	8.45	0.37	0.76	20.81	81.24	0.009	8.19	0.32	0.60	44.65	87.94
